# Integrative Analysis Identifies Multi-Omics Signatures That Drive Molecular Classification of Uveal Melanoma

**DOI:** 10.3390/cancers13246168

**Published:** 2021-12-07

**Authors:** Qianxing Mo, Lixin Wan, Michael J. Schell, Heather Jim, Shelley S. Tworoger, Guang Peng

**Affiliations:** 1 Department of Biostatistics & Bioinformatics, H. Lee Moffitt Cancer Center & Research Institute, Tampa, FL 33612, USA; Michael.Schell@moffitt.org; 2 Department of Molecular Oncology, H. Lee Moffitt Cancer Center & Research Institute, Tampa, FL 33612, USA; Lixin.Wan@moffitt.org; 3 Department of Health Outcomes & Behavior, H. Lee Moffitt Cancer Center & Research Institute, Tampa, FL 33612, USA; Heather.Jim@moffitt.org; 4 Department of Cancer Epidemiology, H. Lee Moffitt Cancer Center & Research Institute, Tampa, FL 33612, USA; Shelley.Tworoger@moffitt.org; 5 Department of Clinical Cancer Prevention, The University of Texas MD Anderson Cancer Center, Houston, TX 77030, USA; gpeng@mdanderson.org

**Keywords:** integrative clustering, iCluster, uveal melanoma, integrative subtype, iSubtype, molecular classification, hypomethylation, hypermethylation, metastasis

## Abstract

**Simple Summary:**

Uveal melanoma (UM) is a heterogeneous disease driven by accumulative alterations at multi-omics levels including genomics, epigenomics and transcriptomics. It is of great clinical interest to identify UM molecular subtypes and subtype-specific biomarkers that could be used for prognosis and targeted therapy. Integrative clustering (iCluster) analysis is a new approach designed to identify cancer integrative subtypes (iSubtypes) and multi-omics signatures that drive molecular classification of cancer. In this study, we performed an iCluster analysis of UM multi-omics data and identified four UM iSubtypes, which formed two major iSubtypes (denoted by M3 and D3) with distinct multi-omics landscapes. We showed that the integrative molecular classification of UM was determined by concordant alterations at multi-omics levels including DNA copy number, DNA methylation, gene expression and somatic mutation. We further derived a gene panel that can be used to classify UM into high- or low-risk groups for metastasis.

**Abstract:**

By iCluster analysis, we found that the integrative molecular classification of the UM was primarily driven by DNA copy number variation on chromosomes 3, 6 and 8, differential methylation and expression of genes involved in the immune system, cell morphogenesis, movement and migration, and differential mutation of genes including *GNA11*, *BAP1*, *EIF1AX*, *SF3B1* and *GNAQ*. Integrative analysis revealed that pathways including IL6/JAK/STAT3 signaling, angiogenesis, allograft rejection, inflammatory response and interferon gamma response were hypomethylated and up-regulated in the M3 iSubtype, which was associated with a worse overall survival, compared to the D3 iSubtype. Using two independent gene expression datasets, we demonstrated that the subtype-driving genes had an excellent prognostic power in classifying UM into high- or low-risk groups for metastasis. Integrative analysis of UM multi-omics data provided a comprehensive view of UM biology for understanding the underlying mechanism leading to UM metastasis. The concordant molecular alterations at multi-omics levels revealed by our integrative analysis could be used for patient stratification towards personalized management and surveillance.

## 1. Introduction

Uveal melanoma (UM), originating from melanocytes within the uveal tract of the eye, is a relatively rare cancer, with a mean age-adjusted incidence of 5.1 cases per million per year in the United States [1]. However, it is the most common cancer of the eye and the second most common form of melanoma after cutaneous melanoma (CM). UM is a highly aggressive disease that often develops distant metastases within a few years after diagnosis, despite successful local treatment of the primary tumors [2]. It preferentially metastasizes to the liver, with more than 90% of metastatic cases showing hepatic lesions [3]. The risk of developing metastases is related to many factors including genetic and transcriptomic features. Genetic features including loss of a copy of chromosome 3 (monosomy 3), mutations in *BAP1* (*BRCA*-associated protein 1) and gain of chromosome 8q were often found to coexist in UM and were associated with high risk of metastasis [4,5,6,7]. In contrast, tumors with two copies of chromosome 3 (disomy 3), gain of chromosome 6p, mutations in *EIF1AX* and *SF3B1* were less likely to metastasize [4,7,8,9]. Currently, no adjuvant systemic therapy has been demonstrated to reduce the risk of metastasis [10]. In a retrospective study of 661 patients diagnosed with metastatic UM over three decades (1982–2011), the median survival time was less than a half year; the one-year survival rate was about 21%, and the three-year survival rate was less than 5% [11].

In an effort to comprehensively characterize UM, The Cancer Genome Atlas (TCGA) research network generated somatic mutation, gene expression (GE), DNA copy number and methylation data for 80 primary UM samples [12]. These multi-omics data are great resources for studying UM molecular subtypes. Based on datatype-specific clustering analysis, TCGA reported datatype-specific UM subtypes in which the UM samples were classified into four copy number subtypes, four methylation subtypes and four gene expression subtypes, respectively [12]. In the datatype-specific subtype analysis, a certain number of omics features (e.g., genes, CpG loci, genomic regions) were first selected and then used for datatype-specific clustering analysis. As a result, different molecular cancer subtypes were generated, and the samples were classified depending on the individually selected features [13]. It is well known that multi-omics data are inherently correlated. For example, DNA amplification and promoter hypomethylation could lead to an increase of gene expression, whereas DNA copy number loss and promoter hypermethylation could result in decrease of gene expression. In the datatype-specific clustering analysis of UM samples, the inherent data structure of the multi-omics data was not integrated for sample clustering. As a result, it misses the opportunity to reveal the integrative cancer subtypes and the correlated multi-omics features that determine the subtypes.

A more advanced approach for molecular classification of cancers is to perform integrative clustering (iCluster) analyses. The iCluster methods are state-of-the-art integrative approaches that can integrate multi-omics data using a joint statistical model for sample clustering and omics feature selection [14,15,16,17]. Specifically, the iCluster methods use low-dimensional latent variables that form a set of “principal coordinates” to capture the inherent structure of multi-omics data. As a result, tumor samples can form clusters in the latent variable spaces. In addition, cluster-driving omics features are identified through variable selection techniques. Although the iCluster methods have been successfully used to characterize a variety of cancers including squamous cell lung cancers, glioblastoma, gastric adenocarcinoma, esophageal carcinoma, sarcoma and bladder cancer, they have not been used to characterize uveal melanoma (UM) [12,18,19,20,21,22,23]. No integrative UM subtypes derived from iCluster analysis of the multi-omics data have been reported, to the best of our knowledge. We hypothesized that a truly integrative analysis might reveal the inherent structure of the multi-omics data, which would eventually determine the intrinsic molecular classification of UM. Therefore, in this study, we aimed to identify UM integrative subtypes (iSubtypes) and compare them with the datatype-specific subtypes. Using UM multi-omics data as an example, we demonstrated that iCluster analysis was able to identify intrinsic UM subtypes and subtype-driving gene expression signatures that had prognostic value in predicting UM metastasis.

## 2. Materials and Methods

### 2.1. Multi-Omics Data Sets

Primary UM samples were collected and analyzed by TCGA using multiple molecular profiling platforms [12]. The TCGA UM multi-omics data (version 2016_01_28) were obtained from the Firebrowse portal (http://firebrowse.org/, accessed on 19 December 2018), which included DNA copy number, DNA methylation, RNA-seq gene expression, and somatic mutation data for 80 UM samples. The multi-omics data were processed by TCGA using the standard bioinformatics pipelines (https://docs.gdc.cancer.gov/Data/Introduction/) and the level-3 of the data were used. Specifically, the DNA copy number levels were measured by log2 ratio segment means of contiguous chromosome segments for each chromosome. The DNA methylation levels of known CpG sites were measured by the beta value, which was the ratio of the methylated probe intensity to the overall intensity (sum of methylated and unmethylated probe intensities). The beta values that had minimum correlation with corresponding mRNA expression were used for the iCluster analysis. The genome-wide GE was measured by RNA-seq and quantified by the RSEM method [12]. The somatic mutations were identified by whole exome sequencing and the annotated somatic variants were used for the integrative analysis. Two microarray GE datasets with accession numbers GSE22138 and GSE44295 were obtained from the GEO database (https://www.ncbi.nlm.nih.gov/geo/, accessed on 21 September 2020). The GSE22138 data set was made of genome-wide GE data of 63 primary UM samples, and the GSE44295 data set was made of genome-wide GE data of 57 primary UM samples and 6 other cell samples [24,25].

### 2.2. Integrative Clustering Analysis

Integrative clustering analysis of the UM multi-omics data was performed using the iClusterPlus method [15]. To perform this analysis, the UM multi-omics datasets were processed to form 4 data matrixes with each row representing a sample and each column representing an omics feature. Specifically, the somatic mutation data were processed to form a binary matrix with 0 indicating wild type and 1 indicating mutation for a gene in a sample. A gene was considered to be mutated if there were in frame deletion/insertion, frame shift deletion/insertion, missense/nonsense/nonstop mutation, RNA, splice site and/or translation start site mutation in its sequence. We filtered out the genes with low mutation rates (<2%) in the 80 samples, which left 116 genes with mutation rates ≥2% used for iCluster analysis. The copy number data were processed to form an 80 × 2632 matrix with rows representing the 80 samples and columns representing 2632 chromosome segments, which were condensed from the original segment means of the continuous chromosome regions by applying the CNregions function of the iClusterPlus package (https://bioconductor.org/packages/release/bioc/html/iClusterPlus.html, accessed on 12 December 2019). The methylation data used for the iCluster analysis were made of an 80 × 4052 matrix with columns representing the top 25% (4052) most variable genes. To better fit the iClusterPlus model, logit transformation of the methylation beta values was performed. The mRNA expression data were made of an 80 × 4776 matrix with columns representing the top 25% (4776) most variable genes. Log2 transformation of the mRNA-seq normalized counts plus 1 were performed to fit the iClusterPlus model. The genes with low mutation rates and expression variation were filtered out because it was highly unlikely that they were the driving force for sample clustering, and it could significantly reduce the computation time. To find an optimal number of clusters and the model that best fit the data, we tested the cluster number parameter K from 1 to 5 and searched 701 lasso parameters for each K. We identified the lasso parameters at which the deviance ratio was the maximum for each K. When K = 4 and K = 3, the Bayesian Information Criterion (BIC) values were the smallest and the second smallest, respectively (Supplementary Figure S1). For a given K, the number of the clusters is K + 1; therefore, 5- and 4-cluster solutions were the two best options. We found that clusters 1, 2 and 3 were almost identical for the 4- and 5-cluster solutions (Supplementary Table S1). The major difference of the 4- and 5-cluster solutions was that cluster 4 (D3.2) of the 4-cluster solution was further divided to 2 smaller clusters that formed the 5th cluster (Supplementary Table S1). Therefore, to avoid overfitting, we proceeded with the 4-cluster solution. Omics features with non-zero slope parameters contributed to the sample clustering.

### 2.3. Classification and Bioinformatics Analysis

To investigate if the expression signature of selected genes (e.g., the differentially expressed genes on chr3, 6 and 8 with absolute expression fold change >2) had prognostic value, we performed classification analysis using the TCGA mRNA data as the training dataset and the other two microarray gene expression data as the testing datasets. In the testing datasets, if multiple probes were used to measure a gene’s expression, we selected the probe with the largest variance to represent the gene. Then, for each sample, the z-score for each gene was calculated and used for the classification analysis. We used the k-nearest neighbor method for the classification analysis, where k = 3 was chosen because the cross-validation error was minimum for both the expression signatures when k = 3 by testing k = 1, 3, 5, 7, …, 31, 33. Gene set enrichment analysis was performed using software GSEA 3.0 (https://www.gsea-msigdb.org/gsea, accessed on 27 July 2018). Gene ontology term enrichment analysis was performed using the clusterProfiler package from Bioconductor (https://bioconductor.org/packages/release/bioc/html/clusterProfiler.html, accessed on 26 October 2020).

### 2.4. Statistical Analysis

Kaplan–Meier method was used to estimate the subtype-specific survival and log-rank test was used to compare the survival curves of the subtypes. Fisher’s exact test was used to evaluate the association between the somatic mutations and the subtypes. The moderated *t*-test implemented in LIMMA package of Bioconductor (https://bioconductor.org/, accessed on 26 October 2020) was used to detect differentially expressed genes between the M3 and D3 subtypes. Genes with undetected expression in more than a half of the samples were removed from this analysis. All the statistical analyses were performed using R 4.0.3 (https://www.r-project.org, accessed on 10 October 2020). P values were two-sided and *p*-value < 0.05 was considered statistically significant for a single comparison. For multiple comparisons, false discovery rate (FDR) was estimated using Benjamini–Hochberg method.

## 3. Results

### 3.1. Integrative Molecular Subtypes of UM

**Figure 1:**
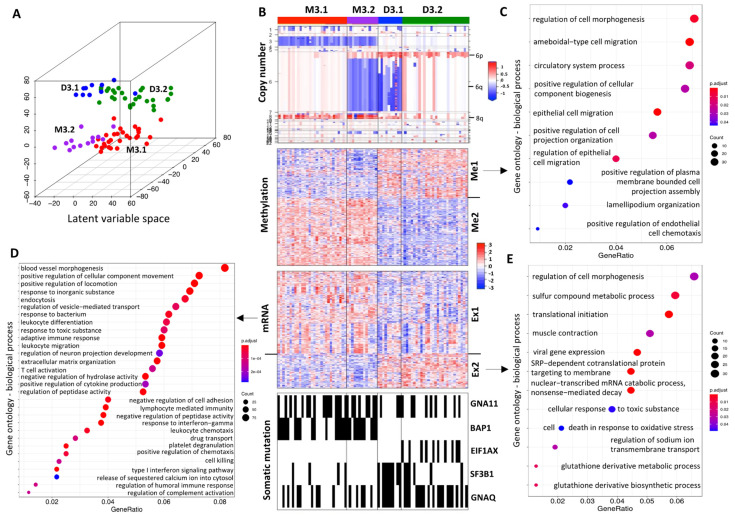
Integrative subtypes of UM. (**A**) TCGA UM samples distributed in the latent variable space (the first 3 principal coordinates) of iCluster. (**B**) Heatmaps of multi-omics features. Copy number: blue, white and red represent potential copy number loss, normal and gain, respectively. Methylation: blue and red represent hypomethylation and hypermethylation, respectively; driver genes (Supplementary Table S4) form two methylation clusters Me1 and Me2. mRNA: blue and red represent low and high expression, respectively; driver genes (Supplementary Table S4) form two mRNA expression clusters E1 and E2. Somatic mutation: samples with mutated genes are indicated by black bars. (**C**) Top enriched biological processes in methylation cluster Me1. (**D**) Top enriched biological processes in mRNA expression cluster Ex1. (**E**) Top enriched biological processes in mRNA expression cluster Ex2.

We identified four UM iSubtypes, which were characterized by distinct molecular patterns across multi-omics levels (Figure 1A,B). The integrative clustering of the UM samples was primarily driven by the inherent patterns of DNA copy number, methylation and mRNA expression (Figure 1B). In UM, it is well known that the most common chromosomal aberration is monosomy 3 (M3, loss one copy of chromosome 3). Strikingly, driven by the iClusterPlus algorithm and inherent multi-omics patterns, the UM samples with loss of chromosome 3 formed a cluster (M3), and the samples with normal chromosome 3 formed another cluster (D3) (Figure 1B, copy number). Therefore, we named the two major iSubtypes M3 and D3 to reflect the most prominent features defined by the status of chromosome 3 (Figure 1B, copy number). Gains of chromosomes 6p and 8q were also frequently observed in UM. The iCluster analysis revealed that, in general, M3 coexisted with 8q gain, and D3 coexisted with 6p gain, while M3/8q gain and 6p gain appeared to be mutually exclusive (Figure 1B, copy number). Based on the status of 6q, the M3 samples were further divided into two subgroups, M3.1 and M3.2, where M3.2 was characterized by loss of 6q. Similarly, the D3 samples were further divided into two subgroups D3.1 and D3.2, where D3.1 was characterized by loss of 6q (Figure 1B, copy number).

The top methylated genes that drove the sample clustering formed two clusters, Me1 and Me2, that had opposite methylation patterns (Figure 1B, methylation). The genes in the Me1 cluster were hypomethylated in the M3 iSubtype, compared to the genes in the D3 iSubtype. In contrast, the genes in the Me2 cluster were hypermethylated in the M3 iSubtype, compared to the genes in the D3 iSubtype. Enrichment analysis of gene ontology (GO) terms showed that the Me1 cluster was primarily enriched with genes involved in cell morphogenesis, migration and their regulation (Figure 1C), while no significant biological process GO terms were found in the Me2 cluster (FDR > 0.05). The top genes in the mRNA data that drove sample clustering also formed two major clusters, Ex1 and Ex2 (Figure 1B, mRNA). In the Ex1 cluster, the top enriched biological processes were involved in cell morphogenesis/movement/migration (e.g., blood vessel morphogenesis, positive regulation of locomotion/chemotaxis/cellular component movement, leukocyte migration/chemotaxis, negative regulation of cell adhesion) and immune network (e.g., T cell activation, lymphocyte mediated immunity, adaptive immune response, leukocyte differentiation, regulation of humoral immune response, cell killing) (Figure 1D); and the genes involved in these processes had a relatively higher expression in the M3 iSubtype compared to the D3 iSubtype. In the cluster Ex2, the top enriched biological processes were involved in cellular metabolism (e.g., sulfur compound metabolic process, glutathione derivative biosynthetic/metabolic process) and protein biosynthesis/metabolism (e.g., translational initiation, SRP-dependent cotranslational protein targeting to membrane, nuclear-transcribed mRNA catabolic process, nonsense-mediated decay) (Figure 1E); and the genes involved in these processes had relatively higher expression in the D3 subtype, compared to the M3 subtype.

The top mutated genes that contributed to the UM sample clustering included *GNA11*, *BAP1*, *EIF1AX*, *SF3B1* and *GNAQ* (Figure 1B, somatic mutation). The M3 iSubtype was characterized by higher frequencies of mutations in *GNA11* (57% vs. 32% in D3, *p* = 0.026, Fisher’s exact test) and *BAP1* (62% vs. 0% in D3, *p* < 0.0001). In contrast, the D3 iSubtype was characterized by higher frequencies of mutations in *EIF1AX* (26% vs. 0% in M3, *p* = 0.00029, Fisher’s exact test), *SF3B1* (37% vs. 9.5% in M3, *p* = 0.0063), and *GNAQ* (66% vs. 36% in M3, *p* = 0.013). It should be noted that *BAP1* and *EIF1AX* mutations were mutually exclusive, and there was only one sample harboring both *EIF1AX* and *SF3B1* mutations among the 27 samples with *EIF1AX* and/or *SF3B1* mutations. In addition, *BAP1*, *EIF1AX* and *SF3B1* mutations concentrated in the M3, D3.2 and D3.1 iSubtypes, respectively (Figure 1B, somatic mutation).

### 3.2. Comparison of UM iSubtypes with Datatype-Specific Subtypes

**Figure 2:**
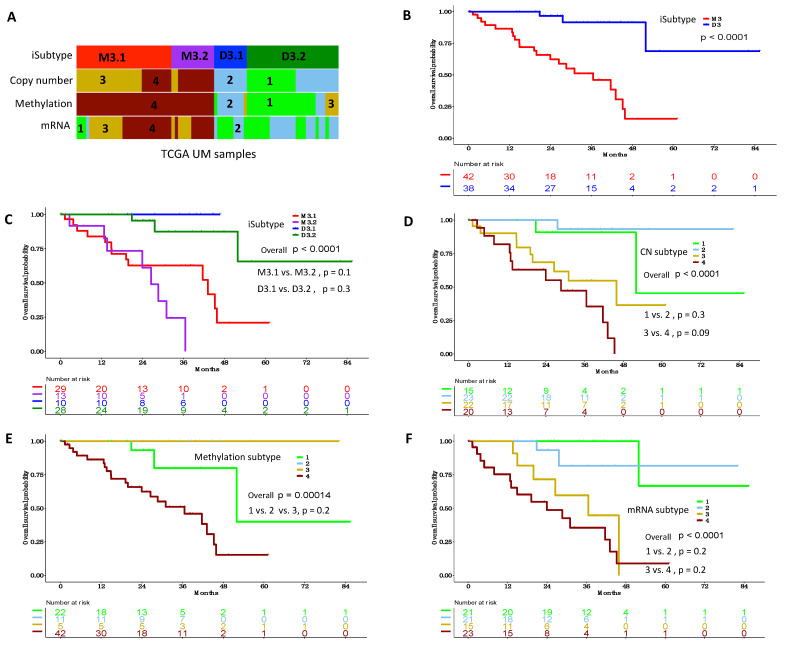
Comparison of iSubtypes with datatype-specific subtypes in the TCGA cohort. (**A**) TCGA UM samples (*n* = 80) colored according to the iSubtypes, TCGA DNA copy number, TCGA methylation, and TCGA mRNA subtypes, respectively. (**B**) Patient overall survival of M3 and D3 iSubtypes. (**C**) Patient overall survival of the M3.1, M3.2, D3.1 and D3.2 iSubtypes. (**D**) Patient overall survival of the TCGA DNA copy number (CN) subtypes. (**E**) Patient overall survival of the TCGA methylation subtypes. (**F**) Patient overall survival of the TCGA mRNA expression subtypes. Log-rank test was used to compare subtype-specific survival curves.

TCGA reported datatype-specific UM subtypes based on DNA copy number, DNA methylation and mRNA expression. Interestingly, the M3 iSubtype was identical to the TCGA methylation subtype 4 and the combination of the TCGA copy number subtypes 3 and 4, and was nearly identical to the combination of the TCGA mRNA subtypes 3 and 4 (Figure 2A). The D3 iSubtype was identical to the combination of the TCGA copy number subtypes 1 and 2 and the combination of the TCGA methylation subtype 1, 2 and 3, and was nearly identical to the combination of the TCGA mRNA subtypes 1 and 2 (Figure 2A). Based on these results, it can be seen that iCluster and datatype-specific analyses basically revealed two major UM subtypes, which were characterized by the chr3 status (M3, D3). However, the iCluster and the datatype-specific approaches differed in further dividing UM samples to smaller subgroups. The M3 iSubtype was significantly associated with a worse overall survival, compared to the D3 iSubtype (Figure 2B, *p* < 0.0001, log-rank test). The significant differences in overall survival among the four iSubtypes were primarily driven by the M3 and D3 classification (Figure 2C, overall *p* < 0.0001, log-rank test), while there was no significant difference in overall survival between the subgroups of the two major iSubtypes (Figure 2C; M3.1 vs. M3.2, *p* = 0.1; D3.1 vs. D3.2, *p* = 0.3, log-rank test). Similar findings were observed in the TCGA datatype-specific subtypes, with significant differences in overall survival primarily driven by the two major subtypes defined by the chr3 status (M3, D3), while overall survival was not significantly different between the subgroups of the two major subtypes (Figure 2D–F).

### 3.3. Differentially Methylated and Expressed Genes between the M3 and D3 iSubtypes

**Figure 3:**
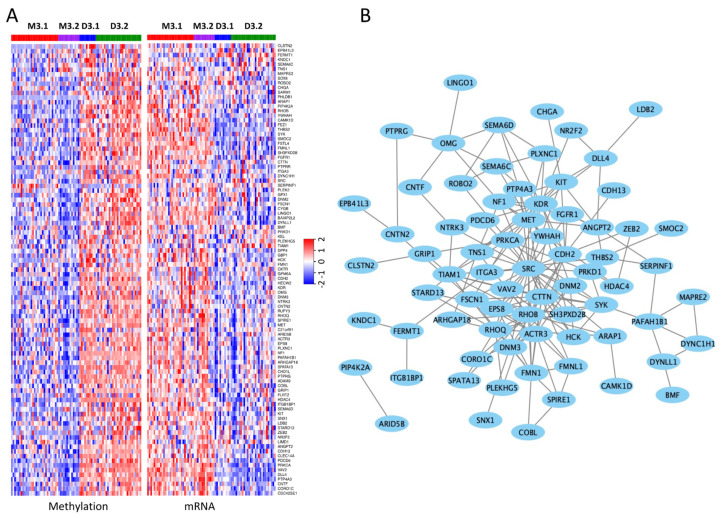
Methylation and expression patterns of genes involved in cell migration, projection and morphogenesis. (**A**) Heatmaps of gene methylation and mRNA expression. The genes in the methylation and mRNA heatmaps are arranged in the same order from top to bottom, as shown at the right side of the heatmaps. (**B**) Protein–protein interaction network derived from STRING database.

The iCluster analysis identified that the genes involved in cell morphogenesis/migration and their regulation were hypomethylated in the M3 iSubtype, compared to the D3 iSubtype (Figure 3A, left panel). As expected, in general, these hypomethylated genes had a higher expression in the M3 iSubtype, compared to the D3 iSubtype (Figure 3A, right panel). The encoded proteins of these genes form a protein–protein association network according to the STRING database [26], where the *SRC* gene appears to be center of the network (Figure 3B). *SRC* encodes a non-receptor protein tyrosine kinase that plays an important role in controlling a variety of biological activities including cell adhesion, migration, transformation and invasion [27]. Other core genes in the network include tyrosine kinase genes (e.g., *HCK*, *MET*, *KDR*, *NTRK3*, *SYK*, *FGFR1*, *KIT*), serine/threonine kinase genes (e.g., *PRKCA*, *PRKD1*), and genes directly involved in cell adhesion and migration (e.g., *CTTN*, *CDH2*, *TNS1*).

**Figure 4:**
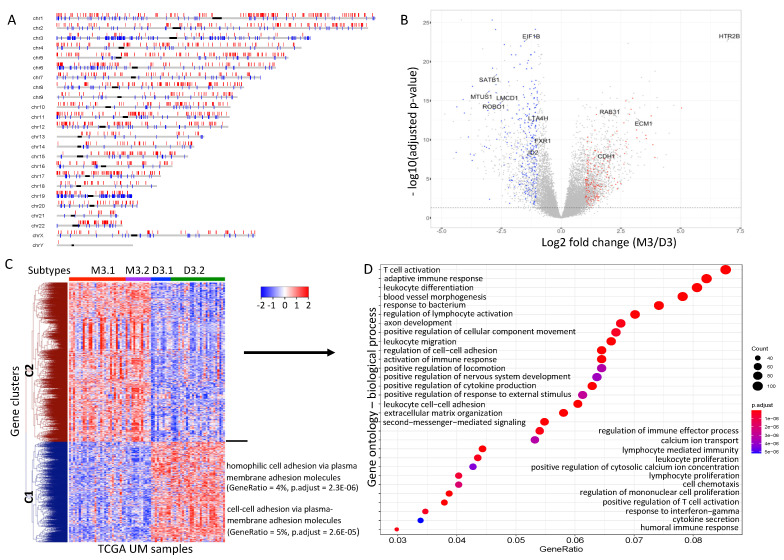
Differentially expressed (DE) genes between the M3 and D3 iSubtypes. (**A**) DE genes distributed on the chromosomes. DE genes are defined as the genes with adjusted *p*-value < 0.05 and absolute fold change of M3/D3 > 2. (**B**) Volcano plot −log10 (adjusted *p*-value) vs. log2 (fold change of M3/D3) of all the genes under differential expression analysis. DE genes with 2-fold lower expression in M3 (vs. D3) are shown by blue dots, and DE genes with 2-fold higher expression in D3 (vs. M3) are shown by red dots. The 12 classical genes with prognostic value are shown on the volcano plot. (**C**) Heatmap of the DE genes (shown in Supplementary Table S4). (**D**) Top 30 most enriched biological processes in gene cluster 2.

Differential expression analysis showed that the gene expression patterns between the M3 and D3 iSubtypes were quite different. Controlling false discovery rate (FDR) at 5%, there were 7681 genes differentially expressed (DE) between the M3 and D3 iSubtypes, in which 2344 genes had at least 2-fold changes (|FC| > 2) in expression. The genomic loci of the DE genes with expression |FC| > 2 are shown in Figure 4A. Consistent with the loss of chr3, about 86% of the DE genes had at least 2-fold decreases in expression in the M3 iSubtype compared to the D3 iSubtype; and consistent with the 8q gain, more than 75% of the DE genes had at least 2-fold increases in expression in the M3 iSubtype (Figure 4A). These observations suggest that the gene expression is generally associated with DNA copy number, but that it is also affected by other factors. Interestingly, the DE genes with expression |FC| > 2 included the 12 genes (*EIF1B*, *FXR1*, *ID2*, *LMCD1*, *LTA4H*, *MTUS1*, *ROBO1*, *SATB1*, *CDH1*, *ECM1*, *HTR2B*, *RAB31*) whose expression has been frequently used for prognostic testing to evaluate the risk of developing metastasis (Figure 4B) [28]. The DE genes with expression |FC| > 2 formed two clusters, C1 and C2, with distinct expression patterns (Figure 4C). Gene cluster C1 (characterized by lower expression of the genes in the M3 iSubtype) was enriched with genes involved in homophilic cell and cell–cell adhesion via plasma membrane adhesion molecules (Figure 4C). Gene cluster C2 (characterized by higher expression of genes in the M3 iSubtype) was mainly enriched with genes involved in immune responses (e.g., T cell activation, adaptive immune response, humoral immune response, activation of immune response, lymphocyte mediated immunity, positive regulation of T cell activation, positive regulation of cytokine production, regulation of lymphocyte activation, lymphocyte/leukocyte proliferation, regulation of immune effector process) and cell morphogenesis/migration/movement (e.g., blood vessel morphogenesis, positive regulation of cellular component movement, positive regulation of locomotion, cell chemotaxis) (Figure 4D).

### 3.4. Pathways and Gene Sets Concordantly Altered in the M3 iSubtype

**Figure 5:**
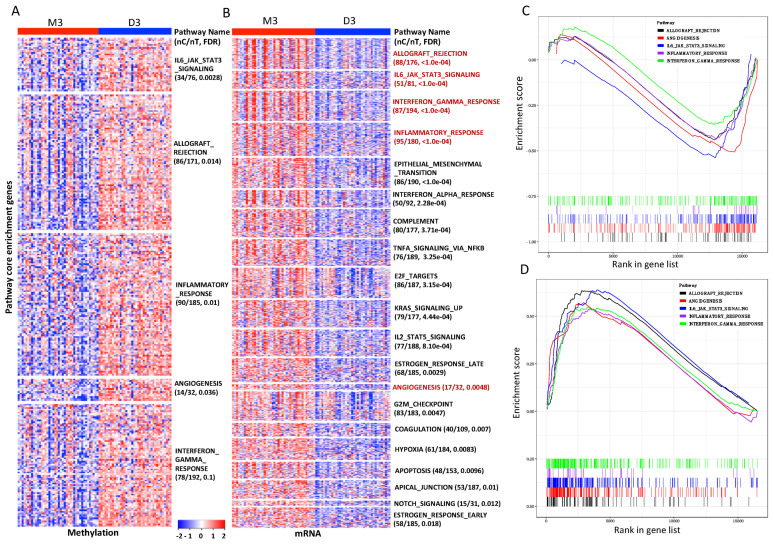
Hypomethylated and up-regulated pathways in the M3 iSubtype of the TCGA UM samples. (**A**) Heatmaps of the core enrichment genes (Supplementary Table S4) of the hypomethylated pathways in the M3 iSubtype. On the heatmap, red and blue represent high and low methylation, respectively. nC/nT: number of core enrichment genes/number of total genes in the pathway. (**B**) Heatmaps of the core enrichment genes of the up-regulated pathways the M3 iSubtype. On the heatmap, red and blue represent high and low expression, respectively. (**C**,**D**) Enrichment plot of the 5 hypomethylated pathways and the corresponding 5 up-regulated pathways, respectively.

To identify the pathways that might be concordantly altered between the M3 and D3 iSubtypes, we performed gene set enrichment analysis (GSEA) of the TCGA methylation and mRNA expression data on the gene sets defined in the Hallmark and Biocarta pathways, which included 50 classical biological pathways and 298 signaling and metabolic pathways, respectively. Pathways including IL6/JAK/STAT3 signaling, allograft rejection, inflammatory response, angiogenesis and interferon gamma response were hypomethylated in the M3 iSubtype (Figure 5A,C). Concordantly, these five pathways were up-regulated in the M3 iSubtype (Figure 5B,D). Interestingly, the top up-regulated pathways in the M3 iSubtype were mainly involved in immune system (allograft rejection, interferon alpha/gamma response, inflammatory response, complement (part of the innate immune system)), signaling transduction (IL6/JAK/STAT3, TNFA signaling via NFKB, KRAS, IL2_STATS, NOTCH), cell cycle (E2F targets, G2/M checkpoint) and tumor progression (epithelial-to-mesenchymal transition, angiogenesis, coagulation, hypoxia). Sixteen of the top 20 up-regulated pathways in the TCGA cohort were also significantly up-regulated in the GSE22138 cohort (FDR < 0.1, Supplementary Figure S2). Pathways including IL6/JAK/STAT3 signaling, interferon gamma and alpha responses were significantly up-regulated in the TCGA, GSE22138 and GSE44295 cohorts (FDR < 0.1, Supplementary Figure S2). GSEA of Biocarta pathways identified five up-regulated pathways with FDR < 0.05 in the M3 iSubtype, which were mainly involved in immune system (CTLA4, CSK, TCR) and tumor progression (SPPA, BAD) (Supplementary Table S2).

To gain insight into the tumor microenvironment in the UM tumors, we investigated if the M3 and D3 iSubtypes were differentially enriched with tumor-infiltrating lymphocytes (TILs). The immunome compiled by Bindea et al. was used for this study, which consisted of 28 immune-cell-specific gene sets representing 28 different immune cell types [29]. The GSEA results revealed that adaptive immune cells including T cells (FDR < 0.001), Cytotoxic cells (FDR = 0.023), T helper 2 (Th2) cells (FDR = 0.024) and Th1 cells (FDR < 0.021) were significantly enriched in the M3 iSubtype, compared to the D3 iSubtype (Supplementary Figure S3), while the innate immune cells such as macrophages and neutrophils were not (FDR > 0.1). This analysis implied that the M3 tumors were infiltrated by T cells, Cytotoxic cells and Th1/Th2 cells in a higher degree than the D3 tumors.

### 3.5. Prognostic Value of Subtype-Driving GE Signature in Multiple Cohorts of Study

**Figure 6:**
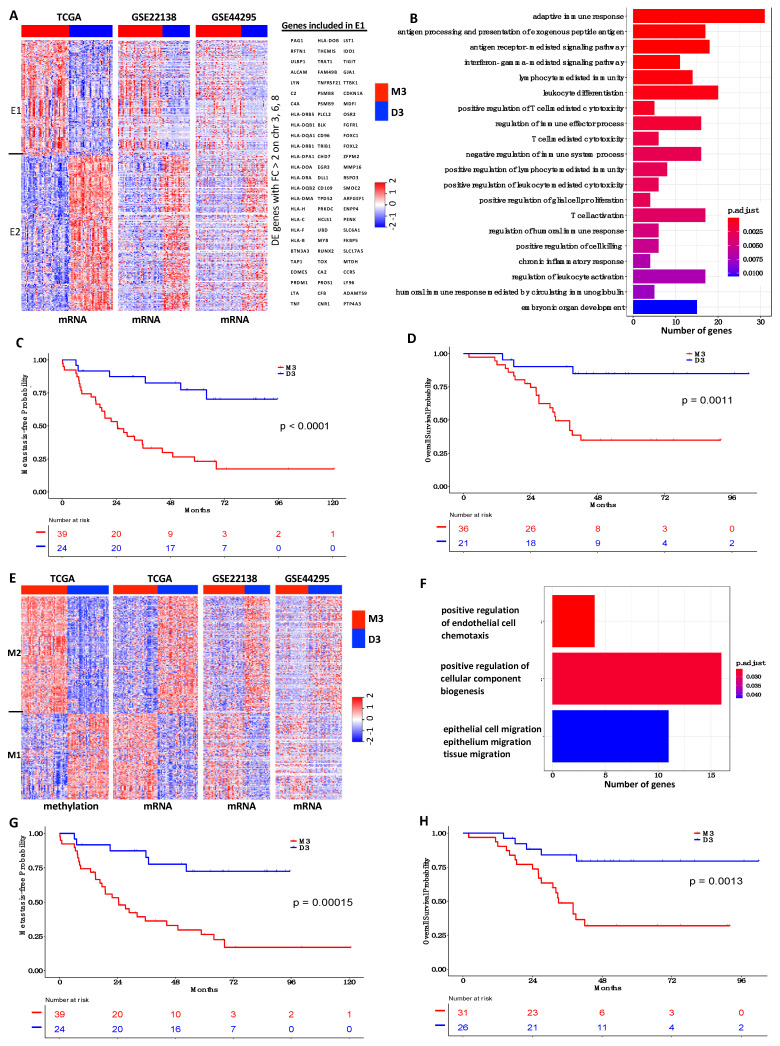
Prognostic power of the iSubtype-driving gene expression signatures. (**A**) Expression heatmaps of the DEG368 in the training (TCGA) [12] and testing (GSE22138, GSE44295) datasets. DEG368 refers to the genes on chr3, 6, 8 with FDR < 0.05 and absolute fold change of M3/D3 > 2 (Supplementary Table S4). (**B**) Top 20 most enriched biological processes in the gene cluster E1. (**C**) Patient metastasis-free survival of the M3 and D3 subtypes defined by the DEG368 expression signature in the GSE22138 cohort. (**D**) Patient overall survival of the M3 and D3 subtypes defined by the DEG368 expression signature in the GSE44295 cohort. (**E**) Expression heatmaps of the DMEG in the training and testing data sets. DMEG refers to the 378 differential methylation and expression genes (Supplementary Table S4). (**F**) Enriched biological processes in the gene cluster M1. (**G**) Patient metastasis-free survival of the M3 and D3 subtypes defined by the DMEG expression signature in the GSE22138 cohort. (**H**) Patient overall survival of the M3 and D3 subtypes defined by the DMEG expression signature in the GSE44295 cohort.

Since the formation of UM iSubtypes was primarily driven by chromosome aberrations on chromosomes 3, 6 and 8 (chr3/6/8) and the differentially methylated and expressed genes (Figure 1B), we investigated whether the related genes could be used to classify UM samples into subtypes with clinical implications. The DE genes on chr3/6/8 with expression |FC| > 2 (denoted by DEG368) formed two clusters, E1 and E2, in which the cluster E1 was enriched with genes involved in various immune responses and their regulation (Figure 6A,B). The iSubtype-driving genes included 378 differentially methylated and expressed genes (DMEG), which formed two gene clusters, M1 and M2 (Figure 6E). The cluster M1 was enriched with genes involved in cellular component biogenesis and cell migration (Figure 6F). To test the prognostic values of these genes, we collected two additional microarray GE data sets with accession numbers GSE22138 and GSE44295 from the publicly available GEO database (https://www.ncbi.nlm.nih.gov/geo/, accessed on 21 September 2020). The GSE22138 data set included microarray GE data of 63 primary UM samples, and the GSE44295 data set included microarray GE data of 57 primary UM samples and six other samples [24,25]. Using the TCGA GE data set as a training set, we classified the samples in the two testing sets (GSE22138, GSE44295) into M3 or D3 subtype based on the DEG368 and the DMEG signatures, respectively. We observed that the GE patterns in the TCGA training dataset were also present in the two testing datasets (Figure 6A,E). Compared to the D3 subtype, the M3 subtype defined by DEG368 signature was associated with a worse metastasis-free survival in the GSE22138 cohort (Figure 6C, *p* < 0.0001, log-rank test), and a worse overall survival in the GSE44295 cohort (Figure 6D, *p* = 0.0011, log-rank test). Very similar results were observed for the M3 subtype defined by the DMEG signature (Figure 6G,H).

In the GSE22138 dataset, 55 samples were evaluated by array CGH or FISH and the chr3 status (disomy, monosomy, partial monosomy) of the samples were provided. Among the 18 chr3 disomy samples, 17 (94%) and 16 (89%) of them were classified as disomy by the DEG368 and the DMEG signatures, respectively (Supplementary Table S3). Among the 32 chr3 monosomy samples, 30 (94%) of them were classified as monosomy by the DEG368 and the DMEG signatures (Supplementary Table S3). These results indicated that both the DEG368 and the DMEG signatures achieved a high sensitivity and specificity in classification of chr3 monosomy and disomy samples. However, four (80%) and five (100%) of the five chr3 partial monosomy samples were classified as disomy by the DEG368 and the DMEG signatures, respectively (Supplementary Table S3). This result indicated that the two GE signatures could not distinguish chr3 partial monosomy from disomy. In addition, for the eight samples with unknown chr3 status, seven (88%) and six (75%) of them were classified as disomy by the DEG368 and the DMEG signatures, respectively (Supplementary Table S3).

## 4. Discussion

We performed iCluster analysis of UM multi-omics data and demonstrated that UM samples could be classified into four iSubtypes that formed two major UM iSubtypes by taking multi-omics data into account. Through iCluster analysis, we showed that the integrative clustering of UM samples was primarily driven by the alterations in chromosomes 3, 6 and 8, as well as differential methylation, gene expression and mutation patterns, which were not revealed by individual clustering analysis as reported by Robertson et al. [12]. By comparing iSubtypes with the DNA copy number, methylation and mRNA-specific subtypes, we found that there were actually two major UM subtypes coinciding with the chromosome 3 status (M3, D3). When these two major subtypes were further divided into smaller subgroups, no significant difference in overall survival was observed in the smaller subgroups (Figure 2). These observations suggest that classifying UM into two major subtypes might be sufficient for personalized management and surveillance in practice.

The iCluster analysis identified *GNA11*, *BAP1*, *EIF1AX*, *SF3B1*, and *GNAQ* as the drivers for the clustering of the UM samples. Interestingly, *GNA11* was mutated at a higher frequency in the M3 iSubtype, while *GNAQ* was mutated at a higher frequency in the D3 iSubtype (Figure 1B). *GNA11* and *GNAQ* are involved in the Gα11/Q pathway, which regulates many biological processes including cell proliferation and growth, and their activating mutations are considered the major drivers of UM carcinogenesis [30,31]. *BAP1* mutation and its loss due to monosomy 3 were associated with high risk of metastasis [6]. However, it is still unclear whether it plays a direct role in UM metastasis. Our integrative analysis identified that genes involved in cell morphogenesis/migration and their regulation were concordantly methylated and expressed in the M3 iSubtypes, which could provide insights into the mechanisms of UM metastasis (Figure 3A). Among the genes involved in cell morphogenesis/migration, *SRC* and *CTTN* were located in the center of protein–protein interaction network (Figure 3B). *SRC* is a proto-oncogene that encodes a non-receptor protein tyrosine kinase that regulates a variety of biological activities such as gene expression, immune response, cell adhesion, migration and transformation [32,33]. *CTTN* encodes a cytoplasmic protein that plays a role in organizing the cytoskeleton and cell adhesion structures of epithelium cancer cells, and is involved in the formation of invasiveness and metastases [34]. *SRC* protein regulates cytoskeletal organization through phosphorylation of cortactin (*CTTN*), which is a critical step for the formation of invadosome and the early metastatic cascade [27]. Protein tyrosine phosphatase type IV A member 3 (*PTP4A3*) was found to be hypomethylated and upregulated in the M3 iSubtype (Figure 3A). *PTP4A3* might play a direct role in promoting metastasis because overexpression of *PTP4A3* in UM cell lines led to a significant increase of cell migration and invasiveness [24]. *PTP4A3* is located in chromosome 8q24.3, a region frequently amplified in M3 tumors. However, it was found that overexpression of *PTP4A3* was not merely a result of 8q amplification [24]. We found that *PTP4A3* hypomethylation might explain its overexpression in the M3 tumors.

The enrichment analysis of the Hallmark pathways revealed that E2F targets and G2/M checkpoint pathways were up-regulated in the M3 tumors. E2F family consists of at least seven transcription factors (*E2F1*–*E2F7*) that play an important role in regulating cell cycle and tumorigenesis [35]. Up-regulation of E2F targets suggests that E2F transcription factors are active, which could promote cells transiting from G1 phase to S phase and facilitate DNA replication. Among the up-regulated genes in the G2/M checkpoint pathways, *CCNB2* and *CDK1* proteins form a complex that directly controls the cell cycle at the G2/M checkpoint (Supplementary Table S4). Up-regulation of *CCNB2* and *CDK1* accelerates tumor cell mitotic progression. The enrichment analysis also revealed that pathways related to tumor progression, including epithelial-to-mesenchymal transition (EMT), angiogenesis and hypoxia, were up-regulated in the M3 tumors. Furthermore, the angiogenesis and EMT pathways were among the top hypomethylated pathways in the M3 tumors, suggesting that DNA methylation might play a role in regulating the gene expression in these pathways. Among the up-regulated genes in the EMT pathway, *TGFB*, *EGF*, *PDGFB*, *ZEB1* and *FOXC2* play important roles in the induction of EMT (Supplementary Table S4). When the epithelial cells of the primary tumors undergo EMT, they lose their cell polarity and cell–cell adhesion and gain enhanced migratory capacity and invasiveness [36]. Up-regulation of the angiogenesis and hypoxia pathways suggest that the M3 tumors tend to grow more rapidly than the D3 tumors, which require a greater blood and oxygen supply. These observations could help to understand the underlying mechanisms of the aggressive nature of the M3 tumors.

Although immunotherapies using monoclonal antibodies against CTLA4, PD1 and PDL1 have been successfully used to treat patients with metastatic cutaneous melanoma resulting in improved patient survival, they have not yet led to significant clinical benefits for patients with metastatic UM [37,38,39]. It should be noted that although *CTLA4*, *PD1* and *PDL1* were expressed in a relatively higher level in the M3 tumors, their overall expression levels were relatively low (Supplementary Figure S5). The enrichment analysis of immune-cell-specific gene sets showed that the M3 tumors were enriched with T cells, cytotoxic cells and Th1/2 cells (Supplementary Figure S3). Usually, tumors with TILs respond better to immunotherapy. However, immunosuppressive microenvironment could limit the antitumor effect of TILs. The enrichment analysis revealed that several immune pathways including IL6/JAK/STAT3 signaling, inflammatory response and interferon gamma response were hypomethylated and up-regulated in the M3 tumors. In the tumor microenvironment, elevated IL6/JAK/STAT3 signaling could suppress antitumor immune response and promote cancer cell proliferation, survival, invasiveness and metastasis [40]. It is well documented that interferons can up-regulate major histocompatibility complex (MHC) molecules I and II. Consistently, we found that the M3 tumors had a higher expression of human leukocyte antigen class I and II molecules than the D3 tumors (Figure 6A). Up-regulation of HLA molecules could help UM cells escape from natural killer (NK) cell-mediated cytolysis [41]. In addition, *TGFB*, *IDO1* and *TIGIT* were expressed in a higher level in the M3 tumors. TGF-β can suppress NK cell activation and function, and *IDO1* and *TIGIT* protein can inhibit T cell and NK cell responses, which might contribute to the development of UM metastasis [42,43,44,45]. Furthermore, the increased number of Th2 cells in the M3 tumors suggest a pro-inflammatory and immunosuppressive tumor microenvironment, which can be detrimental to antitumor immunity [46].

Differential expression analysis revealed that the gene expression patterns were quite different between the M3 and D3 tumors. By integrative analysis of the DNA copy number and gene expression data, we found that the expression patterns of the genes on chromosomes 3 and 8q were consistent with their chromosome status. However, the *HLA* gene cluster located in chromosome 6p had a higher expression in the M3 tumors, although the M3 tumors had a lower copy number than the D3 tumors (Figure 6A). These observations suggest that other mechanisms might be involved in regulating the expression of the *HLA* gene cluster. In addition, we found that the DE genes on chr3/6/8 as well as the iSubtype-driving (differentially methylated and expressed) genes had excellent prognostic power in classifying UM patients into high or low metastatic risk group. These genes appeared to be more consistent in stratifying UM samples, compared to the 12 genes (Figure 6, Supplementary Figure S4). Therefore, these genes could be used for development of clinical test to distinguish patients with low or high metastatic risk.

## 5. Conclusions

In summary, we identified four UM iSubtypes that formed two major iSubtypes with distinct landscapes across multi-omics levels by iCluster analysis. We found concordant alteration at pathway levels among gene expression, DNA methylation and DNA copy number and demonstrated that gene expression signatures can be used as clinically relevant classifiers. The integrative analysis could help to understand the underlying mechanism that leads to UM metastasis.

## Data Availability

The TCGA UM multi-omics data are available at http://firebrowse.org/, accessed on 19 December 2018. The Two microarray GE data sets were obtained from the GEO database (https://www.ncbi.nlm.nih.gov/geo/, accessed on 21 September 2020) with accession numbers GSE22138 and GSE44295.

## Supplementary Materials

The following are available online at https://www.mdpi.com/article/10.3390/cancers13246168/s1, Figure S1: Model fitting parameters of iClusterPlus. Figure S2: Top up-regulated pathways in the M3 subtype of the TCGA, GSE22138 and GSE44295 cohorts. Figure S3: Up-regulated immune gene sets in the M3 iSubtype of the TCGA UM samples. Figure S4: Prognostic power of the 12 gene expression signatures. Figure S5: CTLA4, PDCD1 (PD1), and CD274(PDL1) expression in the M3 and D3 iSubtypes of the TCGA cohort. Table S1: Comparison of 4-cluster solution (K = 3) and 5-cluster solution (K = 4). Table S2: Top up-regulated BIOCARTA pathways in the M3 iSubtype of the TCGA cohort. Table S3: Classification results of the GSE22138 cohort using the expression signatures of the DEG368, DMEG, and the 12 classical genes. Table S4: genes on Figure 1, Figure 4, Figure 5 and Figure 6.
