# Supplementary material for: Integrative Analysis Identifies Multi-Omics Signatures That Drive Molecular Classification of Uveal Melanoma
Source: Cancers (Basel). 2021 Dec 7;13(24):6168. doi: 10.3390/cancers13246168 (PMC8699355; doi:10.3390/cancers13246168)
Supplement: Supplementary file 1 [file cancers-13-06168-s001.zip › cancers-1441059-supplementary.pdf]

cancers

# Supplementary Materials: Integrative Analysis Identifies Multi-Omics Signatures That Drive Molecular Classification of Uveal Melanoma

Qianxing Mo, Lixin Wan, Michael J. Schell, Heather Jim, Shelley S. Tworoger and Guang Peng

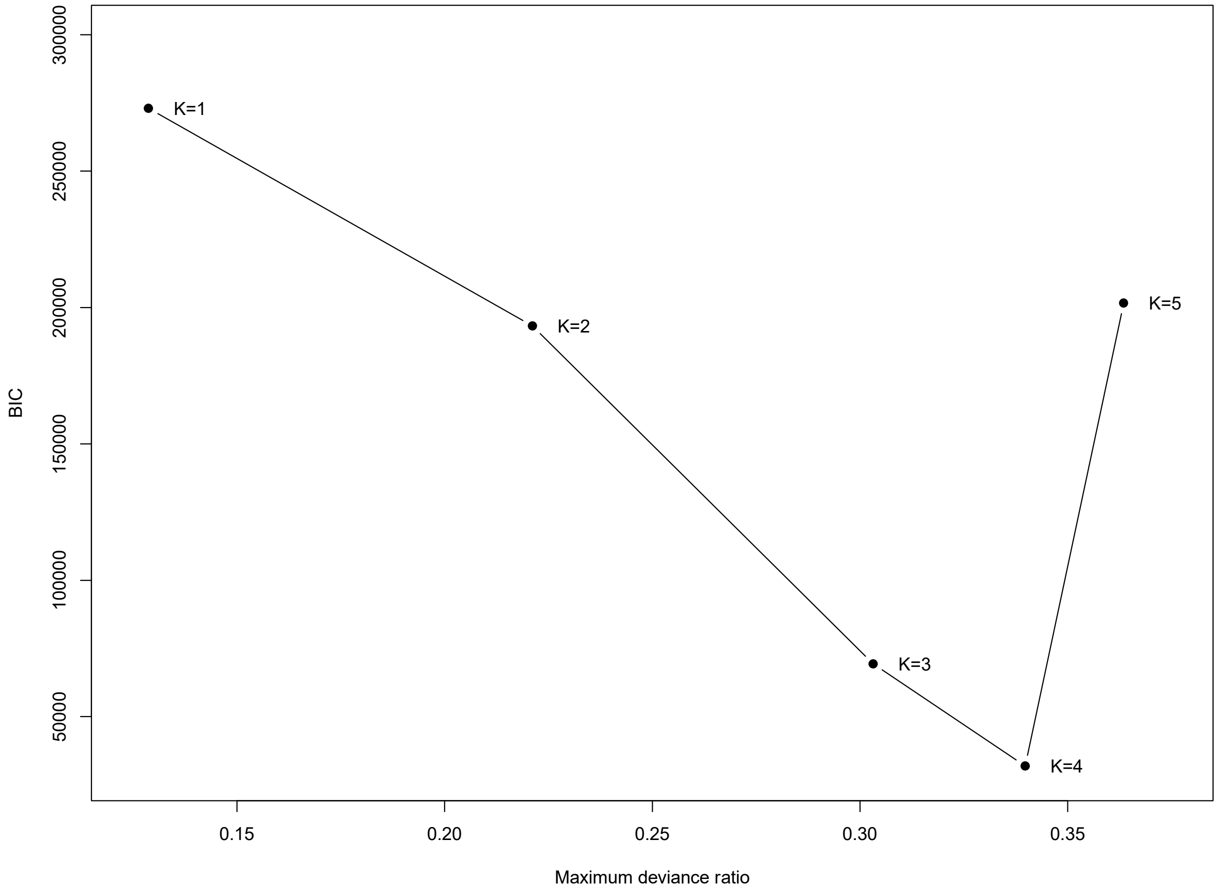


**Figure S1.** Model fitting parameters of iClusterPlus. Bayesian information criterion (BIC) and the maximum deviance ratio for the cluster parameter K=1,2,3,4,5 are shown. For a given k, the samples can be divided into K+1 clusters. Based on the BIC and maximum deviance ratio, K=3 (4 clusters) and K=4 (5 clusters) are two optimal solutions. .

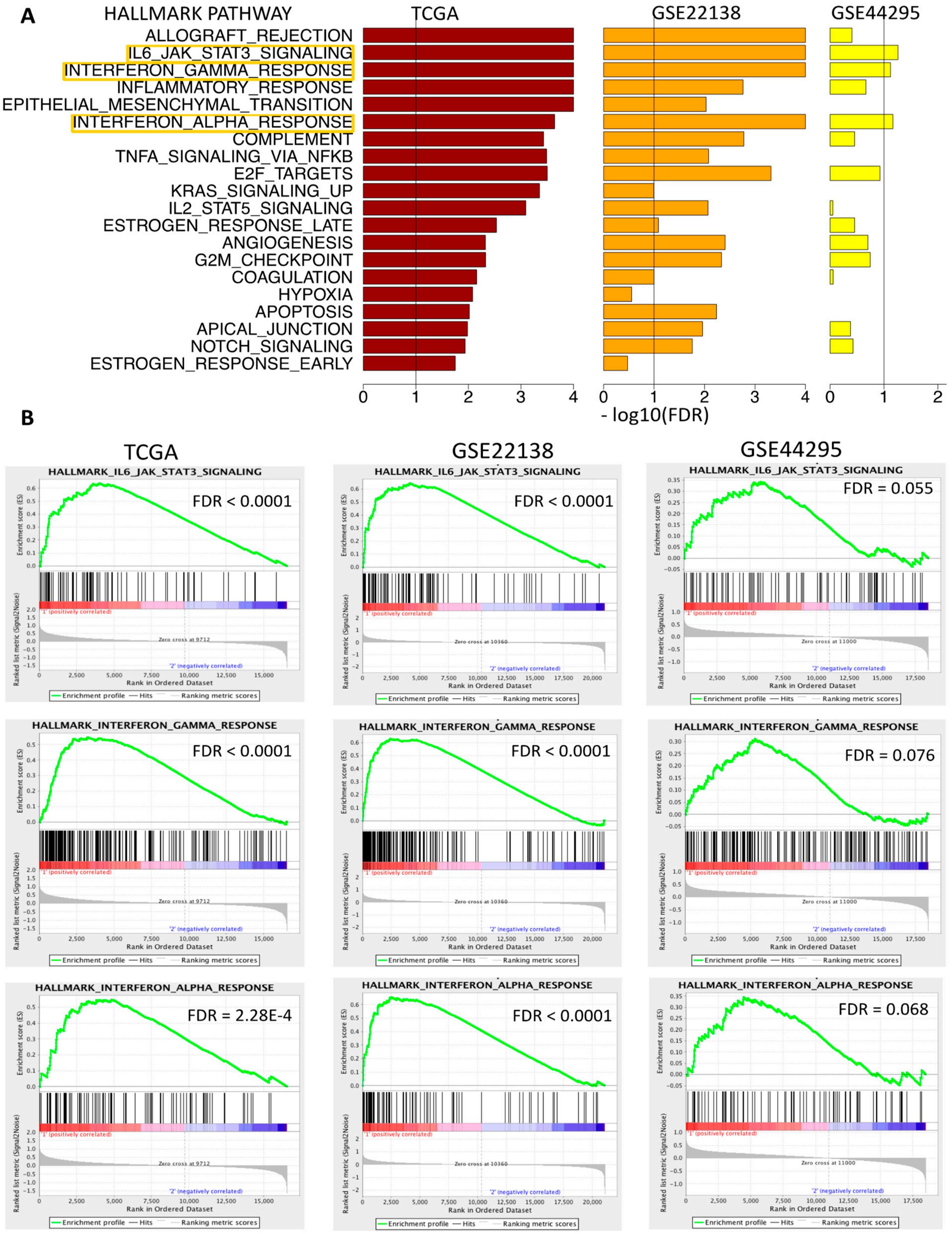


**Figure S2.** Top up-regulated pathways in the M3 subtype of the TCGA, GSE22138 and GSE44295 cohorts. **(A)** Comparison of the significance levels of the top up-regulated pathways. **(B)** Enrichment plots of the most significantly up-regulated pathways.

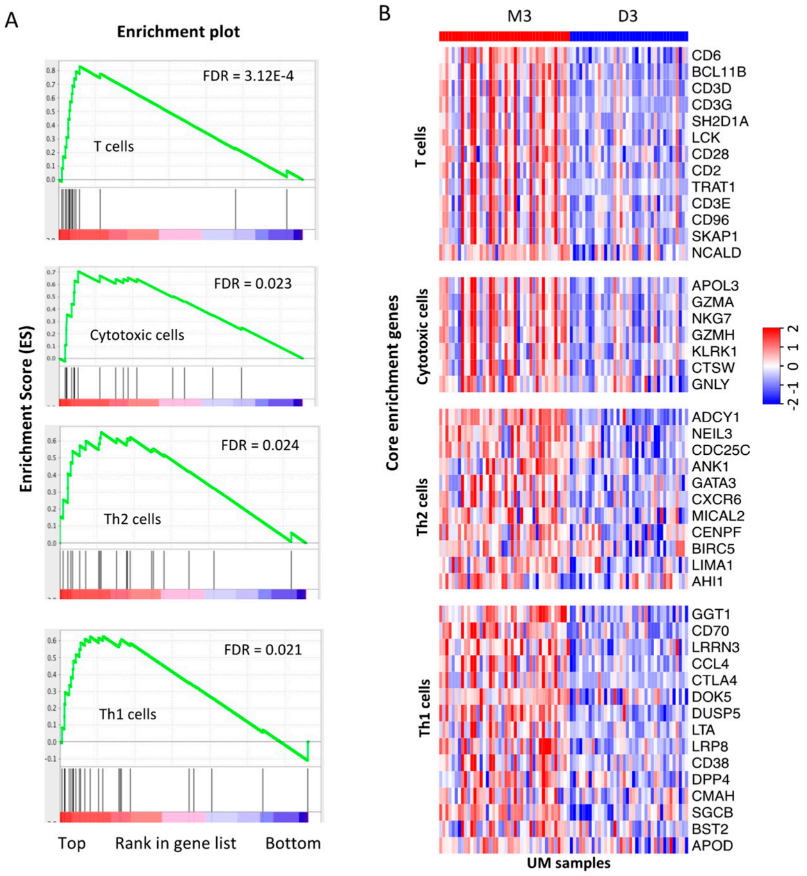


**Figure S3.** Up-regulated immune gene sets in the M3 iSubtype of the TCGA UM samples. **(A)** Enrichment plot of the enriched immune gene sets. Gene-based permutation test was used to calculate the FDR. **(B)** Heatmaps of the core enrichment genes of the up-regulated immune gene sets in the M3 iSubtype. On the heatmap, red and blue represent high and low expression, respectively.

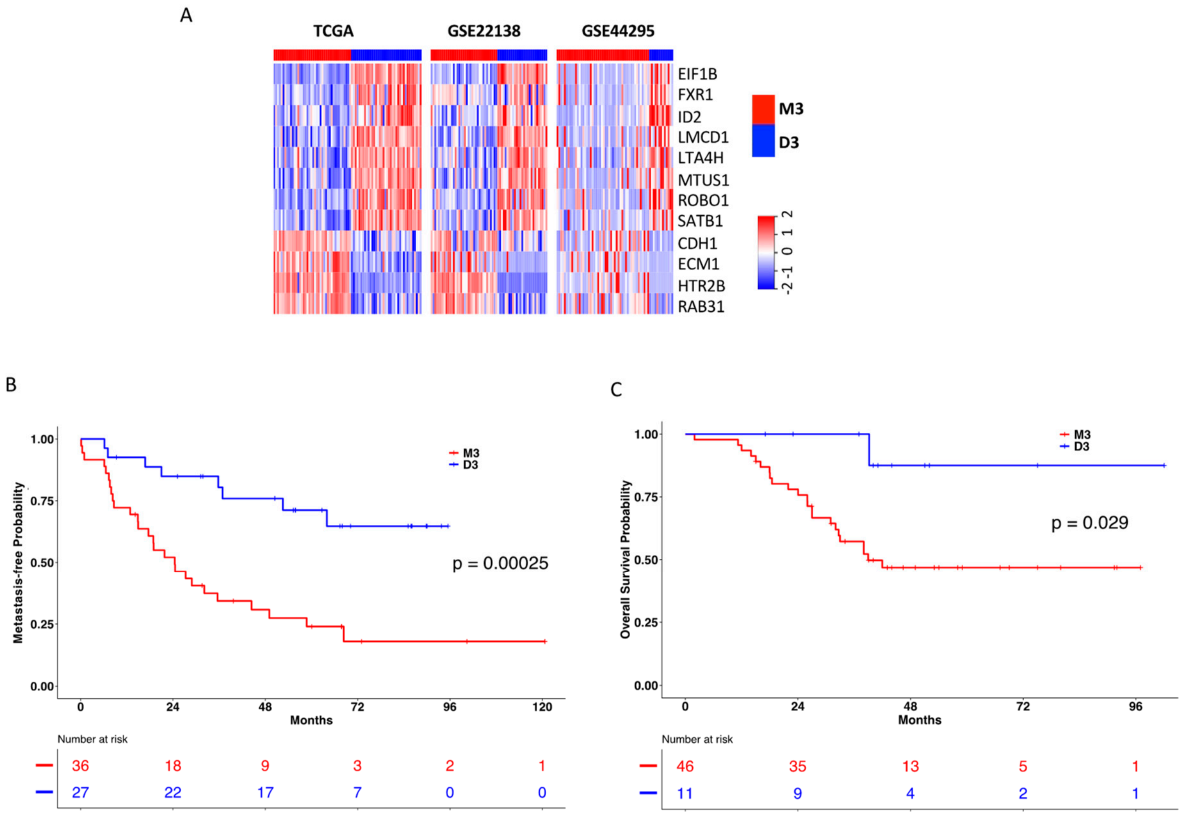


**Figure S4.** Prognostic power of the 12 gene expression signatures. **(A)** Heatmaps of the 12 gene expression of the training and testing data sets. (**B**) Patient metastasis-free survival stratified by the M3 and D3 subtypes defined by the 12 genes in the GSE22138 cohort. **(C)** Patient overall survival stratified by the M3 and D3 subtypes defined by the 12 genes in the GSE44295 cohort.

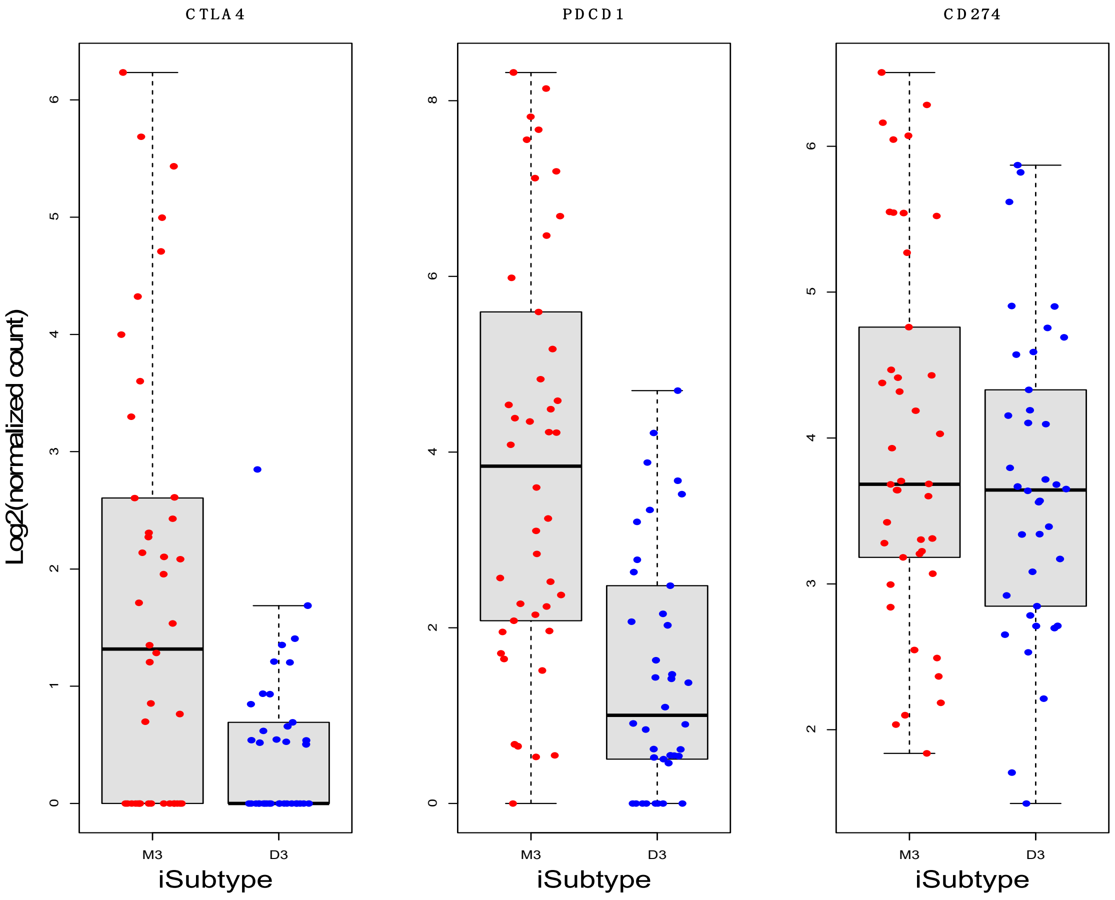


**Figure S5.** CTLA4, PDCD1 (PD1), and CD274(PDL1) expression in the M3 and D3 iSubtypes of the TCGA cohort.

**Table S1.** Comparison of 4-cluster solution (K = 3) and 5-cluster solution (K = 4).

| | **5-cluster solution** | | | | |
|---|---|---|---|---|---|
| **4-cluster solution** | **M.1** | **M.2** | **D.1** | **D.2** | **D.3** |
| M.1 | **29** | 0 | 0 | 0 | 0 |
| M.2 | 0 | **13** | 0 | 0 | 0 |
| D.1 | 0 | 0 | **9** | **1** | 0 |
| D.2 | 0 | 0 | 0 | **23** | **5** |

The values in the table are the numbers of common samples shared by any pair of subtypes in the 4- and 5-cluster solutions.

**Table S2.** Top up-regulated BIOCARTA pathways in the M3 iSubtype of the TCGA cohort.

| **BIOCARTA Pathway Name** | **Description** | **SIZE** | **ES** | **NES** | **p-value** | **FDR** |
|---|---|---|---|---|---|---|
| CTLA4 | The Co-Stimulatory Signal During T-cell Activation | 19 | 0.79 | 2.01 | 0.0000 | 0.0021 |
| CSK | Activation of Csk by cAMP-dependent Protein Kinase Inhibits Signaling through the T Cell Receptor | 19 | 0.79 | 2.00 | 0.0000 | 0.0012 |
| SPPA | Aspirin Blocks Signaling Pathway Involved in Platelet Activation | 15 | 0.75 | 1.81 | 0.0005 | 0.0313 |
| BAD | Regulation of BAD phosphorylation | 23 | 0.67 | 1.76 | 0.0014 | 0.0490 |
| TCR | T Cell Receptor Signaling Pathway | 44 | 0.57 | 1.75 | 0.0004 | 0.0494 |
| INTRINSIC | Intrinsic Prothrombin Activation Pathway | 16 | 0.70 | 1.72 | 0.0041 | 0.0601 |
| EDG1 | Phospholipids as signalling intermediaries | 22 | 0.65 | 1.71 | 0.0036 | 0.0538 |
| PAR1 | Thrombin signaling and protease-activated receptors | 18 | 0.68 | 1.71 | 0.0047 | 0.0517 |

| | | | | | | |
|---|---|---|---|---|---|---|
| INFLAM | Cytokines and Inflammatory Response | 15 | 0.71 | 1.69 | 0.0038 | 0.0620 |
| TOB1 | Role of Tob in T-cell activation | 16 | 0.68 | 1.66 | 0.0086 | 0.0798 |
| CHREBP | ChREBP regulation by carbohydrates and cAMP | 16 | 0.66 | 1.61 | 0.0140 | 0.1261 |
| IL2RB | IL-2 Receptor Beta Chain in T cell Activation | 37 | 0.54 | 1.59 | 0.0078 | 0.1381 |
| NKT | Selective expression of chemokine receptors during T-cell polarization | 20 | 0.62 | 1.59 | 0.0192 | 0.1329 |
| IL12 | IL12 and Stat4 Dependent Signaling Pathway in Th1 Development | 19 | 0.61 | 1.54 | 0.0244 | 0.1975 |
| NKCELLS | Ras-Independent pathway in NK cell-mediated cytotoxicity | 16 | 0.63 | 1.54 | 0.0288 | 0.1856 |
| MPR | How Progesterone Initiates the Oocyte Maturation | 20 | 0.59 | 1.52 | 0.0318 | 0.2091 |
| NO2IL12 | NO2-dependent IL 12 Pathway in NK cells | 15 | 0.63 | 1.51 | 0.0360 | 0.2114 |
| CCR5 | Pertussis toxin-insensitive CCR5 Signaling in Macrophage | 17 | 0.60 | 1.50 | 0.0366 | 0.2091 |
| EICOSANOID | Eicosanoid Metabolism | 22 | 0.57 | 1.50 | 0.0333 | 0.2000 |
| ATM | ATM Signaling Pathway | 19 | 0.59 | 1.50 | 0.0361 | 0.1927 |

SIZE: number of genes in the pathway; ES: enrichment score of GSEA; NES: normalized enrichment score; p-value: raw p-value; FDR: adjusted p-value for multiple comparison.

**Table S3.** Classification results of the GSE22138 cohort using the expression signatures of the DEG368, DMEG, and the 12 classical genes. DEG368: differentially expressed genes on chr3, 6, 8 with FDR < 0.05 and absolute fold change of M3/D3 > 2; DMEG: 378 differentially methylated and expressed iSubtype-driving genes.

| **Chr3 status** | **Subtypes predicted by** | | | | | |
|---|---|---|---|---|---|---|
| | **DEG368** | | **DMEG** | | **The 12 genes** | |
| | **M3** | **D3** | **M3** | **D3** | **M3** | **D3** |
| disomy | 1 | 17 | 2 | 16 | 1 | 17 |
| monosomy | 30 | 2 | 30 | 2 | 29 | 3 |
| NA | 7 | 1 | 7 | 1 | 6 | 2 |
| Partial monosomy | 1 | 4 | 0 | 5 | 0 | 5 |
